# Current Surgical Therapy of Locally Advanced cSCC: From Patient Selection to Microsurgical Tissue Transplant. Review

**DOI:** 10.3389/fonc.2021.783257

**Published:** 2021-12-07

**Authors:** Tito Brambullo, Gian Paolo Azzena, Paolo Toninello, Giuseppe Masciopinto, Alberto De Lazzari, Bernardo Biffoli, Vincenzo Vindigni, Franco Bassetto

**Affiliations:** Clinic of Plastic Surgery, Department of Neurosciences, Padua University Hospital, Padua, Italy

**Keywords:** locally advanced disease, microsurgery, non-melanoma skin cancer, oncoplastic, reconstructive surgery, SCC, skin oncoplastic surgery, squamous cell carcinoma

## Abstract

Among the non-melanoma skin cancers (NMSC) the squamous cell carcinoma (SCC) is one of the most challenging for the surgeon. Local aggressiveness and a tendency to metastasize to regional lymph nodes characterize the biologic behavior. The variants locally advanced and metastatic require wide excision and node dissection. Such procedures can be extremely detrimental for patients. The limit of the surgery can be safely pushed forward with a multidisciplinary approach. The concept of skin oncoplastic surgery, the ablative procedures and the reconstructive options (skin graft, pedicled flap, microsurgical free flap) are discussed together with a literature review.

## Introduction

### Definition of Locally Advanced Cutaneous SCC

In Caucasians, skin squamous cell carcinoma (cSCC) is the second most common type of skin cancer, accounting for approximately 20% of all non-melanoma (NMHC) skin cancers (1).

The definition of “locally advanced” cSCC (lacSCC) is ambiguous, it includes tumors that are not more amenable to surgery or radiotherapy, or those who require a multidisciplinary approach because of their size or clinical implications (2).

While the former cannot be successfully treated with surgery, the latter may have the last chance of cure through an aggressive surgical procedure.

Several parameters have been associated with higher risk of CCS development and subsequently worse prognosis.

Histological features include perineural invasion, poorly differentiated grade, acantholytic subtype, spindle or desmoplastic, and vertical tumor thickness > 2mm (3).

Instead, the clinical parameters are the location (ear, median face), diameter > 2 cm and the recently positive re-excision margin has been shown to be an independent risk factor.

Regarding of tumor size and thickness, respectively defined as the maximum diameter of the SCC and the maximum vertical distance between the tumor outer surface and the deeper cell nest, both of them are clearly related to increased risk of local recurrence and distant metastasis (4).

In contrast, primary tumor operability and tumor thickness of < 6mm were correlated with improved overall survival (5).

However, the two parameters mentioned above do not appear to accurately describe the salient features of a locally advanced SCC.

A large, thick and almost entirely exophytic SCC may have a remarkable size, but does not represent an insurmountable challenge for a dermatosurgeon qualified in plastic reconstruction techniques.

Conversely, a medium size tumor with an increased in-depth invasion, that spreads well beyond the subcutaneous fat layer, can require extremely aggressive resection with the sacrifice of functional structures like vessels, nerves and bone, thus causing disfiguring outcome and functional impairment.

So the Breslow measurement, expressing the mere cancer thickness, does not perfectly match with the anatomical tumor depth, so may not represent a valid parameter for defining an SCC “locally advanced” (6).

Limited to oral SCC, some studies correlated the tumor depth even with the risk of regional lymph node metastasis (7), advocating the necessity of elective regional dissection for tumors > 5 mm in thickness.

This argument solidifies anatomical depth as a predictive factor, on the basis of which a skin SCC might also be considered “advanced”.

Therefore, in relation to the eligibility to surgery of an SCC, the concept of radial extension in the 2-dimensions plane must be replaced with the concept of a 3D space, including the anatomical depth in the evaluation of the real tumor magnitude.

Therefore, the SCC guidelines should include the assessment of growth in depth as well as the diameter of the tumor during the preoperative assessment.

Cutaneous squamous cell carcinoma represents the most common type of a rare female malignancy, the vulvar cancer (8).

Vulvar cancer is often associated with human papillomavirus (HPV) infection and usually affects young women, although HPV-independent SCC most likely affects older women.

The classification of vulvar cancer was revised by the International Federation of Gynecology and Obstetrics (FIGO) in 2014 (9).

The FIGO committee stated that the stromal invasion (defined as the measurement of the tumor from the epithelial–stromal junction of the adjacent most superficial dermal papilla to the deepest point of invasion) and the extension to adjacent anatomical structures play a role in pushing the stage in a higher level with worse prognosis.

So, an advanced vulvar cSCC could be defined by the size (> 2 cm), or if it extents to almost one among of the following urethra, anus and vagina. Recurring vulvar FCS can also be considered “advanced” if it poses a serious local management problem (10).

Squamous cell carcinomas may also occur on the surface of the male genitals.

In the AJCC Staging, 8th ed. 2018 (11), T1 corresponds to a tumor limited to the most superficial layers according to the anatomy of the region (gland, foreskin or shaft).

The perineural invasion contributes to T1 separation in a and b, and the vertical growth to deeper layers like corpus spongiosum and corpus cavernosum pushes the stage forward (from T1 to T2 and from T2 to T3, respectively), accordingly to an increased risk of metastasis and worse prognosis.

Thus, a penile cSCC, that extends deeper than the cutaneous envelope border, can be defined advanced despite the radial size.

### Definition of Metastatic cSCC to Regional Lymph Nodes

In presence of regional nodal metastasis the cutaneous SCC is defined metastatic (mcSCC) (1), but the absence of distant metastasis still permits the operability in selected cases.

The association of any T with regional nodal involvement may be stage III or IVA according to AJCC 8^th^ Ed. 2018 (12).

There is still debate about the appropriate role of surgery in the treatment of a regional metastatic cSCC. In a retrospective study Ch’ng et al. (13) report the grade of differentiation as the only primary tumor factor significantly associated with disease-specific survival.

Other parameters such as clear resection margins, tumor size and thickness, do not seem to have any real impact on the specific survival of the disease in the metastatic population.

Therefore, in mcSCC, surgery may be useful in controlling local disease rather than affecting overall survival, and lymph node exploration is an intrinsic component of the procedure.

## Patient Selection

### The Multidisciplinary Tumor Board Discussion

The initial presentation of a large (>2 cm) cSCC fixed to a deep plane invariably requires the surgeon to determine whether the tumor is operable or not.

That issue should not be addressed only by a dermatosurgeon, but would need the support of other specialists, due to anatomical structures to be resected and/or a complex reconstruction to be accomplished.

Another problem may be the recurring cSCC, which, after previous surgery with R1 o R2 margins, would still be considered recutable by a more extensive excision.

The multidisciplinary tumor board has proven to be effective in better cancer staging, and tumor management can differ in about 10% of cases, compared to what a single specialist would do (14).

However, the concrete impact of the multi-disciplinary approach on outcomes such as improved quality of life (QOL) or overall survival or disease-free survival has not yet been proven.

Undoubtedly the benefit of such preoperative evaluation is the possibility of management of the most complex clinical scenario (15), when the patient overall evaluation is required regarding surgery feasibility and the use of multimodal treatments.

### Risk and Performance Assessment

A detailed clinical history review and a comprehensive physical assessment of the patient are mandatory prior to any difficult surgery.

Comorbidities, previous treatments, age and disability can have a significant impact on the final outcome of a surgical procedure in cancer patients.

The specialist has to keep in mind the potential side effects and complication due to these factors throughout the perioperative time, and recognize whether symptoms or organ dysfunction are imputable to cancer treatment or some other cause (16).

The most widely used perioperative score is the Eastern Cooperative Oncology Group/World Health Organization Performance Status (ECOG/WHO PS), that is employed both for short/medium term overall survival (17), and as a prognostic factor predicting extended length of stay after cancer surgery (18).

However, the ASA score appears to be a higher performing score with respect to 90-day postoperative survival (17).

Recently, some criticisms about ECOG have raised, pointing out that the one-dimensional nature of the tool and the assessment by the physician causing intrinsic subjectivity, make the score inadequate for oncologic tailored treatments (19).

Moreover, accurate discrimination between patients before and during wound healing appears to have a considerable impact on QOL and global outcomes (20).

In terms of functional impairment before surgery, the Barthel score is generally recorded at hospital admission.

A pre-existing functional disability at the time of diagnosis seems to have a significantly lower survival rate and indicates a need for interventions to improve prognosis (21).

The transition to the recording of the dimensions of fragility, multimorbidity and functional status was therefore recommended as part of standard clinical practice.

These results provide a valuable insight into global cancer treatment and encourage health professionals to plan for the early launch of rehabilitation programs to improve functional status.

### The Imaging

Indications for radiology imaging of lacSCC are the need to detect invasion of adjacent/deep anatomical structures and the presence of regional/distant nodal involvement.

Computed tomography (CT) is the cornerstone of assessing the soft tissue extent of the tumor, bone invasion, and nodal metastases.

Pros of CT scans are the high definition of cortical bone surface, if bony invasion, and the detection of abnormal lymph nodes (not smaller than 1.0 cm in size), that can be precisely localized and identified as metastatic (22, 23).

The drawbacks are the need for iodinated contrast for better definition, which can cause or increase kidney failure in at-risk individuals.

Moreover, CT is less sensitive than magnetic resonance (MR) for intracranial diseases, perineural tumor spread, and soft tissue imaging such as muscle fascia or fat.

In selected scenarios, like temporal or orbital invasion, is often useful a combined preop study with CT and MR for optimal planning, due to the presence of different in density tissues and layers (22).

MR scans also allow fine assessment of the extent of tumor invasion in soft tissue (22, 23), while clear guidelines lack for radiologic imaging of patients with presumed perineural spread, it is generally agreed that high-resolution MR is the most sensitive imaging modality available (24).

The disadvantages of MR are incompatibility with implanted ferromagnetic devices and the need to stand still during the examination to avoid motion artifacts. This can prevent the acquisition of patients unable to remain immobile for essential tremors or Parkinson’s disease, which is not uncommon in elderly patients.

Staging of lymph nodes can be performed in different modalities, undoubtedly ultrasound (US) is the least expensive, painless and non-invasive. US does not require immobilization of the patient, and has no risk of adverse reaction to contrast agents (23).

When suspicious lymph nodes are identified, a fine needle aspiration biopsy (FNAB) with US guidance can be used for sampling, given its higher sensitivity and specificity than conventional FNAB (23).

High-frequency US has been used for assessment of the size and extent of primary non-melanoma skin cancers, including depth invasion of the primary tumor (25, 26), but the need of special instrument and dedicated training precludes the systematic application.

The main disadvantage of the US is its intrinsic dependence on the operator, which can greatly influence the sensitivity and accuracy of the exam (23).

In occult metastasis detection the positron emission tomography (PET) plays a main role, the combination with CT is more sensitive in detecting nodal and distant tumor metastases than each modality separately (23).

Fields of application of PET-CT are detection of distant visceral metastases and occult adenopathy, it is successfully used in monitoring of tumor response to therapy, and surveillance of tumor recurrence.

In the latter scenario, this type of imaging is especially useful as it is able to detect local metabolically active relapse in areas with surgically modified anatomy (22).

The major drawback of PET CT is the false positives identified in areas of infection or acute/chronic inflammation that are not related to the neoplastic process. In addition, given the high metabolic demand of the brain, PET CT is not useful in assessing brain metastases, often requiring a separate MRI scan.

### Timing of Surgery

Radiotherapy is an effective nonsurgical therapy available to patients with NMSC.

Cutaneous SCC is radiosensitive and most small cSCC treated with definitive RT exhibit complete remission and extremely low local recurrence (<5%).

Usually, younger patients are given hypofractional radiotherapy for consecutive days over a 4-5 week period to obtain the best long-term outcome.

In older patients instead, the preferred fraction size is higher in order to reduce the overall time of treatment within 2-3 weeks, because of poor performance status that often contraindicates extended daily treatment (27).

Late cutaneous side effects following hypofractioned RT have been documented and potential skin necrosis should not be ruled out; therefore, a fractioned regimen is optimal to reduce this disadvantage.

In the presence of a locally advanced cSCC, deemed resectable, usually a wide excision to obtain R0 margins and subsequent reconstruction is preferable according to high risk of metastasis or debilitating disease progression within 3 months (28).

Following surgery, adjuvant radiation therapy should be avoided unless there is an extended N1 or N2 disease, or if there are “close” or R1 resection margins.

The combination of surgery and radiotherapy can be extremely effective in treatment of lacSCC developed in high risk areas for perineural invasion like ear, orbit and mid-face location (29, 30).

In a different scenario, the lacSCC can be considered unresectable in first instance, so definitive radiotherapy with curative or palliative intent may be administered.

An incomplete response to radiotherapy, or a tumor enlargement, may pose an indication to a salvage surgical procedure after irradiation.

The decision to implement multimodality treatment (postoperative radiotherapy) or salvage treatment (surgery after irradiation) is based on a careful multidisciplinary evaluation (31).

Aside from the well-established benefits in cancer treatment, it has been shown that preoperative radiotherapy increases the risk of postoperative complications (27).

Early radiation lesions consist of an acute inflammatory response and tissue vessel friability that can significantly affect the success of immediate surgery.

Conversely, the fibrosis process induced after RT can increase over time, negatively impacting the success rate of delayed reconstruction (32).

Previous irradiation may cause serious wound-healing problems, and immediate reconstructive procedure after tumor resection may be compromised as well by subsequent adjuvant radiotherapy.

For high rates of reconstruction failure when performed on an irradiated bed, post-operative radiotherapy has been suggested whenever possible (33).

When clinical circumstances require RT prior to surgery, the procedure appears more likely to be successful if carried out within 6 weeks, later the complication rate increases (33).

Another retrospective study on 217 free grafts in 199 patients compares the RT effects on tissues before and after surgery (34).

The conclusion is that the vascularization of the grafted bed decreases continuously according to the total dose and time after radiation treatment.

A time interval of 4 to 6 weeks following RT prior to surgery is then indicated to be preferable.

In a review of 2009 (35), about the effects of RT on microsurgical head and neck reconstruction, several confounding factors have been highlighted like dose of radiation, type of radiation, intensity modulated radiotherapy (IMRT) and variations in fractionation.

It has been hypothesized that all these variables may affect the outcome of reconstructive surgery, in addition to having an impact on oncology therapy.

Controversial studies have been conducted on the incidence of wound complications following concomitant chemo therapy (35).

As with radiation therapy, the timing of chemotherapy is a factor in the onset of complications.

Chemotherapy in the 2 weeks prior to or 1 week following surgery appears to cause more healing complications (36).

Although the effects of chemotherapy are transient, when added to radiation treatment, they tend to have a more severe impact on wound healing.

In a retrospective analysis (37) of 131 patients affected by advanced SCC of head and neck, 38 (29%) underwent 50 surgical procedures after chemoradiotherapy.

Complications were observed in 4 (11%) of the 38 patients and 5 (10%) of the 50 procedures.

Overall, the rates of major and minor complications across all interventions were 6% and 10% respectively.

Furuta et al. (38) instead reported major complications occurring in 8/34 (23.5%) of the group that received chemoradioteraphy before surgery, and 5 of the 8 (62.5%) required additional reconstruction surgery.

Recently Suzuki et al. (39) investigated the different rate of complications, surgical site infection (SSI), and survival in salvage surgery for patients treated by platinum-based chemoradiotherapy (Plat-CRT) or cetuximab-based bioradiotherapy (Cet-BRT).

They demonstrated that patients with Cet-BRT were significantly more associated with the presence of SSI (P < 0.01) and grades IIIb–V in the Clavien–Dindo classification (P < 0.01) used for rating the adverse event gravity.

Moreover, the results demonstrate the significant association between patients with Cet-BRT and older age in good agreement with results previously published by other authors.

All the studies mentioned above are characterised by limitations such as the study design and a small number of subjects.

Despite lack of robust statistical results and although the complications rate increase, there’s agreement to provide anyway a surgical salvage operation to this group of patients, as a last chance, in presence of local recurrence after chemoradiotherapy protocols.

## Tumor Resection

### The Limits of the Ablative Surgery

A lacSCC is a high risk tumor, so the trend is to widen the excision margins respect the low risk ones to decrease local recurrence rate.

It is also important to keep in mind that the metastatic potential of a primary cSCC is independent of the local treatment approach (40).

Recently, the European consensus group (41) suggested a range of 6-10 mm safety margins for cSCC with high risk factors, but pointed out how a specific recommendation on the clinical safety margins cannot be given, because of the lack of consistent reports supporting its independent prognostic value.

The margins width may vary in relation to tumor and patient characteristics, but the opportunity to reduce the extent of resection for aesthetic and functional issues is not clearly mentioned, unlike the specific deviations for special anatomic locations provided for primary site melanoma surgical therapy (42).

This can be explained by the more aggressive biological behaviour of cSCC at the primary site compared to melanoma and the consequent higher risk of recurrence.

In this respect, the current literature is inconsistent, given the lack of randomized trials, and it is not possible to provide conclusive results as regards the superiority of a determined surgical approach to the primary tumor (40).

Physical examination of the lesion with manual palpation and stretching with its surrounding area provides a quick assessment of the extent of involvement.

In spite of this, the actual extent of the lesion may still be vastly underestimated (43).

A not invasive preoperative method to plan more appropriate resection of soft tissue margins is the high frequency ultrasonography, that allows measurement of the 3-dimensional size of tumor with a relevant grade of precision (44).

The findings so far seem encouraging, but some limitations sound evident.

A primary, well defined, small in dimension tumor is objectively easy to examine with US, but in the presence of a large local recurrence surrounded by scarred tissue, that invades the deeper planes modifying the anatomical structures, the accuracy of such measurement appears less reliable.

When bone invasion is suspected, a preliminary study with computed tomography is the best support to calculate the entity of bone resection.

Often lacSCC requires detailed evaluation both the bone and the soft tissues, so the combination of CT scan and MR offers a wide spectrum of information that may allow a precise planning of resection.

This approach is extremely important in head and neck surgery, where imaging is not just used as a pre-operative assessment, but guides the operator throughout the procedure.

The impact of predetermination of excision margins on oncologic outcome has been carefully reported by Pu et al. (45), that compared the preoperative measurement of resection with pathology findings in computer assisted head and neck surgery.

As a rule, they adopted a distance of 15 mm from the bone invasion limit and a distance of 10 mm from the soft tissue involvement.

According to the NCCN Guidelines, surgical margins were classified as ‘clear’ (≥5 mm), ‘close’ (< 5 mm) and ‘positive’ (carcinoma *in situ* or invasive carcinoma at the margin of resection), in relation to the closest distance of resection margin extrapolated from the pathology reports.

More than 80% of the resection margins were clear of invasive tumors and all the bone margins were negative, so they concluded that predetermined surgical margins do not compromise oncological safety.

Main limitations were the small number of cases, the impossibility to determinate the “close” bony margin, due to the necessity of decalcification of the specimen, and the retrospective study design.

### Intra-Operative Margins Assessment

Clinical circumstances and tumor characteristics can prevent fine preoperative planning, and even the most careful imaging has some limitations as well.

The intra-operative margin assessment may be an option to avoid these disadvantages, but each tissue requires a dedicated methodology.

In a remarkable review Rosenthal et al. (46) presented the available optical imaging strategies for intraoperative soft tissue margins assessment.

Optical imaging uses light emitted from a light source (xenon or laser) to magnify the unique properties of tissues with or without optically labelled targeting agents administered.

It allows for real-time feedback providing cancer-specific detection as opposed to peripheral tissue alterations associated with solid tumors.

However, use of these video-assisted surgical techniques necessitates of low ambient light environment and limits the surgeon’s tactile feedback and 3-dimensional tumor visualization, critical in guiding oncology resections in open surgery.

In conventional surgery, a useful method for intra-operative assessment of soft tissue margins is the frozen section.

The surgeon performs the specimen collection, that is immediately processed by the pathologist through marking, freezing and cutting several sections of the specimen at variable distance (1 to 4 mm), then receives a feedback (47).

The question is how reliable is frozen section analysis (intraoperative) respect the standard protocol for formalin-fixed paraffin embedded tissue (postoperative).

A confounding factor is the specific frozen section processing, that can be the so-called “bread-loafing”, thicker slices cut sequentially from the frozen specimen, or the complete circumferential and peripheral and deep margin assessment (CCPDMA), a more time-consuming procedure but with very thinner slices, and so more accurate (48).

Other limits are the sampling or interpretation errors of the specimen.

Due to that, the reports in literature are controversial finding a varying concordance of frozen section and definitive paraffin embedded examination ranging from 80% to 91% (49, 50), thus some have abandoned its use (51).

Factors that may contribute to increase the false negative rate are poorly differentiated subtype, lymphovascular invasion, and perineural invasion (50), frequent histology features in lacSCC.

Instead, there is currently no feasible practice for intraoperative bone margins assessment, due to time required for decalcification of the specimen.

Limitations to this approach lie in the necessity of concrete amount of both cortical and cancellous bone to be examined, the use of tools to obviate the irregularity and hardness of the bony slice, and the contamination by blood cells and bone dust.

The majority of reported results are satisfactory, but technical limitations precluded them from routine clinical application.

In a study of 2014 Nieberler et al. analyzed the intraoperative cytological assessment of the bone resection margins (ICAB) in patients with oral SCC, they attested the technique as reliable and suitable for routine clinical use (52).

In relation to the resection margin status defined by final histology, ICAB provided 80% sensitivity (95% CI, 28-99) and 97.5% specificity (95% CI, 86-99) with 95.5% accuracy.

The results are promising, but a dedicated technical device for brushing the bones and the correct timing of the operating room and pathology process are key to performing the intraoperative procedure.

### The Anatomical Structures to be Saved

With the oncology goal of radical tumor resection, surgery planning must take into account the anatomical structures to be preserved for functional and aesthetic problems.

Randomly planned excision may be effective in the treatment of cancer, but may be detrimental to the patient’s self-esteem, resulting in complaints and frustration.

The wide range of pre-operative exams allows in most cases, even the most complex ones, a realistic anticipation of which tissues should be replaced, repaired or saved.

Often the most challenging areas where lacSCC can develop are face and head region, hand and genitalia (53), so under these circumstances a precise reconstructive plan goes with the oncology procedure.

As mentioned before in this article, in literature there’re not yet a clear indication when it is safe and recommendable to deviate from widen the resection margins in order to preserve as much as possible a very significant part of the body, and a frank and open discussion with the patient on pros and cons is mandatory.

A number of accounts concern about technical solutions to obviate to the impasse (54–56), but they are mainly case or retrospective reports, so it is impossible to draw any robust conclusions.

In a retrospective study on 179 male patients Prodromos et al. (57) found that a limited radical SCC excision with clear margins less than 5 mm did not appear to affect primary oncological control in a high-demanding area like the penile surface.

Local recurrence did not seem to have a negative impact on overall survival, while it was associated with lymphovascular invasion and higher tumor stage and grade.

### En Bloc Resection Versus Micrographically Controlled Surgery: An Open Question

Two different approaches in the eradication of a locally advanced SCC are viable: an en bloc resection, elsewhere named wide large excision (WLE) or standard excision (SE), or a microscopically controlled surgery (MCS), usually referred as Mohs surgery (MMS).

As mentioned above, these two approaches differ not only in the technique of tumor excision, but also in the histological processing.

The first procedure is followed by a delayed specimen examination, usually prepared through the “bread-loafing” technique; the second requires an immediate analysis of multiple frozen slices (another variant, called 3D histology, introduces the paraffin embedded slice fixation).

As a result, the planning chosen by the surgeon affects the methodology adopted by the pathologist.

In consideration of the topic, the locally advanced cSCC, the practice may probably regard a large, thick and invasive tumor or/and relapsing, more than a primary, small and well define one; so the risks of not-free margins and local recurrence are much higher.

The European interdisciplinary consensus guideline on invasive cSCC has stated that cSCC with high-risk factors should be excised with a clinical safety margin of 6-10mm or by MMS/MCS (41).

This statement is based on a number of studies in favor of the superiority of MCS respect standard excision in accuracy and less rate of false negative margins.

One of the most quoted publications, by van Lee et al. (58), is a retrospective cohort study of 579 patients with cSCC treated with MMS or SE, where it is demonstrated a lower recurrence risk of cSCC of the head and neck after MMS (3%) than after SE (8%) during a median follow-up of 5 years.

The results are suggested to be correlated to smaller portion of the excision margin histologically reviewed with SE, so increasing the risk of a false negative result and, consequently, of an misdiagnosis of incomplete cSCC excision.

Several limitations affect that study though, the retrospective design and the impossibility to determine tumor features (depth growth, perineural/lymphovascular invasion and differentiation), risk stratification of patients and disease-specific deaths.

Chren et al. (59) conducted a prospective cohort study of 1174 consecutive patients with primary NMSC, the difference in recurrence rates between standard excision and Mohs surgery was 1.6% during a median follow-up time of 7.4 years.

The results indicated that the two treatments did not differ significantly in preventing local recurrence.

The literature seems unanimous on estimating the MCS superior to standard wide excision in preventing false negative margins and thus local recurrence but this may partially due to a patients selection bias.

Breuninger et al. (40) pointed out that the local recurrence higher rates for WLE and bread-loafing histology may be correlate to the intrinsic features of tumor, usually larger, thicker and higher-risk respect to the ones selected for MCS.

This observation is supported by the clinical practice, in presence of a large and invasive tumor a microscopically controlled surgery would take several hours to be accomplished, given the size of the specimen and the number of margins to be processed.

To reduce the total duration of the procedure, the Mohs surgery lab must be close to the operating room (60), complete with basic equipment needs cryostat, staining equipment and a microscope.

Obviously, specialized lab staff are needed to process the samples.

More, when also bone invasion has to be intraoperatively defined, histology requires different strategies and tools (i.e., cytology) according to soft or hard tissue to be processed, if not, the overall diagnostic power of MCS will inevitably decrease.

Another issue related to adopting the MCS as a standard practice is whether the benefits are related to costs.

Some advocated the advantage of avoiding a potential second surgery for a local recurrence (61), others complained of inadequate reimbursement policies (62).

All these drawbacks make the MCS practicable only in a few selected cases, and not as a routine procedure.

A large, prospective, randomized trial focusing on the prognostic value of WLE and MCS is still missing, making it impossible to draw definitive conclusions.

### Primary Site Management

A single, well defined, cutaneous SCC has been object of numerous studies and the surgical treatment is established in several national consensus groups (63–66), the European international guidelines on invasive squamous cell carcinoma of the skin (41, 67) provide an excellent update on the state of the art.Evidence-based recommendations with high strength of consensus are enunciated about the surgical treatment of SCC primary site and safety margins, although the latter has an inferior level of evidence, because the independent prognostic effect of high-risk factors has not been consistently reported.

A supposed deviation from that would be necessary when a lacSCC develops on a special location, such the preauricolar or periorbital regions (53), but a multidisciplinary surgical approach and a proper operative setting allow to observe the evidence-based guidelines in the majority of cases.

Several simultaneous cSCC or a single invasive cSCC surrounded by various actin keratoses can develop in a single area of the body, the called field of cancerization (68).

The proximity of distinct lesions, even of varying degrees of invasion and differentiation, may exclude the possibility of clear large margin resection.

In addition, there are conditions that predispose to the development of skin cancer, such as genetic alterations and induced immunosuppression; in the affected patients, the scalp, the H-zone and the dorsum of the hands are the most likely locations for other cSCCs in the future.

In these situations the surgery must address the entire cluster of multiple cSCC (41), not only the single locally advanced SCC.

On one side this radical approach permits to get free margins, even if close, and on the other ensures healthy surrounding tissue for a better wound healing.

Usually, excision requires reconstructive surgery, whose complexity may vary depending on the extent and depth of the sample, the segment of the body involved and the specific characteristics of the patient.

Patients can also benefit from multimodal treatment with preoperative or postoperative use of topical agents for local control of resection margins (69).

The size and the deep invasion of a large lacSCC (> 5 cm in diameter) may characterize an extreme case of surgical treatment.

The patient’s good general conditions and the absence of distant metastases may make it possible to consider the feasibility of surgical therapy with radical intention, otherwise meaningless and extremely dangerous.

Literature harbors a wide range of reports documenting the successful treatment of giant squamous cell carcinoma of the skin (70–73), affecting the full-thickness skin envelope and involving the underlying parenchymal organs such as throat, larynx, lung, brain and so on.

A preoperative discussion, the most thorough ever, with the patient is mandatory on realistic expectations in terms of perioperative risk and overall survival.

Albeit technically feasible, extreme procedures can cause patient death for a number of reasons, in addiction the prognosis still remains poor within few months.

Compassionate motives, while commendable, should not influence a rational assessment of the patient.

Surgery may play a role even in the advanced and metastatic cSCC.

Whenever possible, palliative care should be offered without preconceived ideas in terms of opportunity and cost-saving policy.

Case-specific reasons may justify an aggressive and complex procedure for transient or partial recovery, which can greatly benefit the patient’s quality of life over the remaining period (74).

## Lymph Node Management

### Sentinel Lymph Node Biopsy (SLNB)

Worldwide the sentinel node biopsy (SLNB) is largely employed for detection of occult lymph node metastasis of skin cancer.

The technique requires a dynamic lymphoscintigraphy within 24 hours before surgery with injection of a radioisotope at tumor primary site, that consents the intraoperative detection of the first lymph node draining the specific body area with a gamma probe. Alternatively, at the time of surgery a sub-cutaneous injection of blue dye at the primary tumor site will allow the detection of sentinel node (SLN) by staining (75).

The reason to perform SLNB is that earlier detection of occult nodal disease may increase survival or otherwise positively impact the local disease management.

In the AJCC staging system (12) the regional lymph node involvement is considered the worst prognostic factor in cSCC, so the surgical biopsy of sentinel node represents an important staging tool.

Several studies show that cSCC with nodal metastases is still curable, so beyond the staging goal, SLNB may have a curative intent (76).

SLNB has a high sensitivity and a negative predictive value for cSCC (sensitivity 79%, negative predictive value 96%) (77), thus more reliable than conventional imaging (CT and MRI).

At the time of diagnosis, the estimated prevalence of SLN involvement varies considerably in literature, ranging from 7.9% (78) to 21% (76).

The discrepancy may lie in the patients stratification by the risk, because the criteria used to define this parameter differ considerably among the studies and this is a serious limitation for the interpretation of results (78).

In a prospective observational study involving 653 consecutive patients (79), no regional metastasis was observed in patients with Breslow depths less than or equal to 2mm.

The prevalence of metastasis was attested at 4% for patients with a depth ranging from 2.10 to 6 mm and 16% for those with a depth of more than 6 mm.

After a the multivariate analysis, the Breslow depth was the most important predictor of regional metastasis together with tumor diameter and ear location.

In another review (78), no positive SLN was observed in patients with a depth of less than 2mm.

So, it seems that the probability of a positive SLNB increases with the Breslow thickness, especially if it’s more than 6 mm and in association with tumor diameter of more than 2 cm, that are the meaningful features defining a locally advanced cSCC.

The question is whether early detection of occult lymph node metastasis through SLNB impacts the disease-free or the overall survival.

In a retrospective study focused on 720 locally invasive cSCC (thickness > 5 mm) (80), of which 150 underwent to SLNB, 90.9% of all patients developing locoregional metastases showed tumor-free sentinel lymph nodes.

Distant metastasis resulted in 1.58% of patients in the SLNB group and in 1.75% of patients in the observation group (p = 0.898).

Therefore, the results did not support any advantage in local disease control and overall survival in SLNB patients.

Given the serious limitations of the few studies available, no definitive conclusion may be made about the effective role of SLNB in advanced cSCC, further randomized trials are necessary, that compare control groups of patients with comparable high-risk tumors who do not undergo SLNB.

### Regional Lymph Node Dissection

When node metastasis of lacSCC are detected with SLNB or during the preop examination and imaging, there is indication to remove the lymph nodes of the corresponding anatomical region.

The independent prognostic value of lymphadenectomy in relation to overall survival is uncertain, but its role in local disease control is evident.

To date, an elective neck dissection is intended to be radical but, same time, to spare the anatomical structures that do not harbor lymph nodes, and whose resection may cause severe functional impairment.

These structures to be preserved vary according to the different areas of the body.

For the head and neck, the lymph nodes are grouped in six levels according to Robbins (81), to which few other unusual lymph node locations can be added.

Removal of all six levels is not always required due to the lymphatic pathway of head and face drainage.

The selective (partial) lymphadenectomies are classified in base of which levels encompass, thus providing an effective treatment through a less demanding procedure.

The lymph nodes levels to be removed are chosen regard to the primary site of cSCC development, but the second and third level and often the fourth one are included in the resection most of the time, because the contiguity with the internal jugular vein, the anatomical terminal of all the lymphatic pathway.

**Figure 1:**
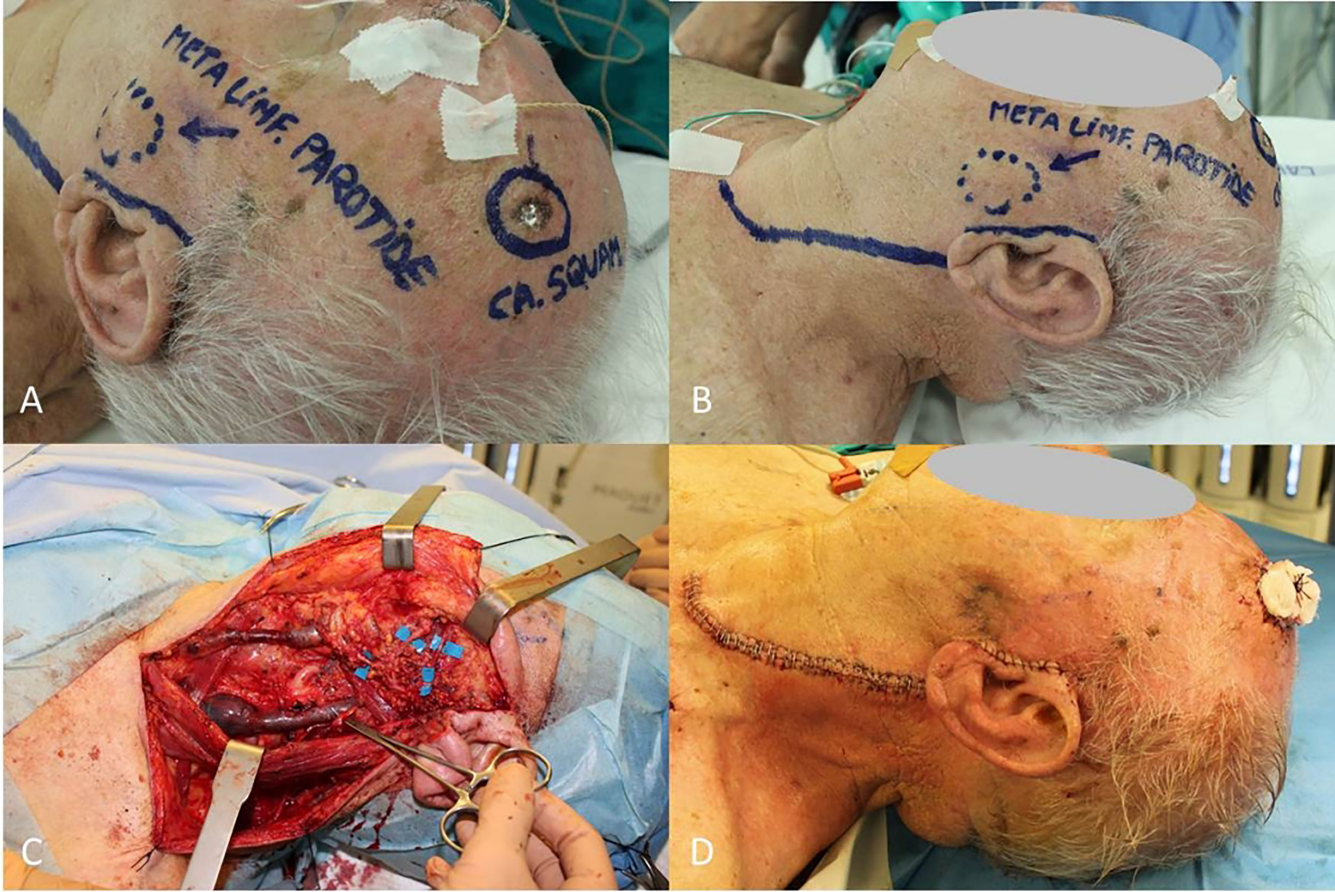
**(A)** cSCC of frontotemporal region; **(B)** Nodal metastasis located in parotid; **(C)** Neck and parotid dissection complete with sparing of internal jugular vein, sternocleidomastoid muscle, accessory nerve and all the branches of facial nerve; **(D)** Final result.

Accessory nerve, sternocleidomastoid muscle and internal jugular vein are intended to be spared unless directly involved by tumor invasion (the so-called functional lymphadenectomy) (**Figure 1**).

In case of parotidectomy, as completion of tumor resection or neck dissection, the terminal branches of the facial nerve should be carefully dissected and spared whenever possible (82).

Intraoperatively is reasonable a change of surgical plan in consideration of macroscopic tumor invasion of one of the above mentioned structures (83).

In upper limb and upper trunk cancer surgery the corresponding lymph nodes are harbored in the axilla.

According to Berg (84), the armpit can be divided in three distinct levels.

The first, most superficial, is burden by the lateral edge of pectoralis major muscle and posteriorly by the edge of latissimus dorsi muscle, the second underlies beneath the pectoralis minor muscle and the third, the deepest, is in contiguity with the superior land mark the axillary vein, that follows up to the cross with the subclavian muscle tendon.

All three levels are generally included in resection, no selective lymphadenectomy is recommended, because of the proximity and continuity of the lymph nodes (41).

The axillary artery and vein, the brachial plexus, the long thoracic nerve of Bell and the thoracic pedicle of the latissimus dorsi should be saved from accidental damage.

No functional impairment is usually appreciable after the procedure, but some reported an occasional postoperative lymphedema affecting the upper limb in about 8-10% of cases.

Immediate physiotherapy and elastic arm dressing may help reduce discomfort.

Instead the groin lymph nodes are to be removed if the mcSCC developed at lower limb, lower trunk and genitalia.

They can be roughly divided into superficial and deep in terms of localization respecting the femoral vein.

The anatomical boundaries of groin are superiorly the inguinal ligament, laterally the edge of the sartorius muscle and medially the edge of the long abductor muscle (Scarpa’s triangle).

Anatomically, the lymphatic drainage path follows the femoral vein, then the external iliac vein, so that even the external iliac fossa and the obturator fossa can be affected by nodal involvement.

Interestingly, contrary to the guidelines on the treatment of melanoma, there is no specific indication to extend lymph node removal to extraperitoneal level (41).

**Figure 2:**
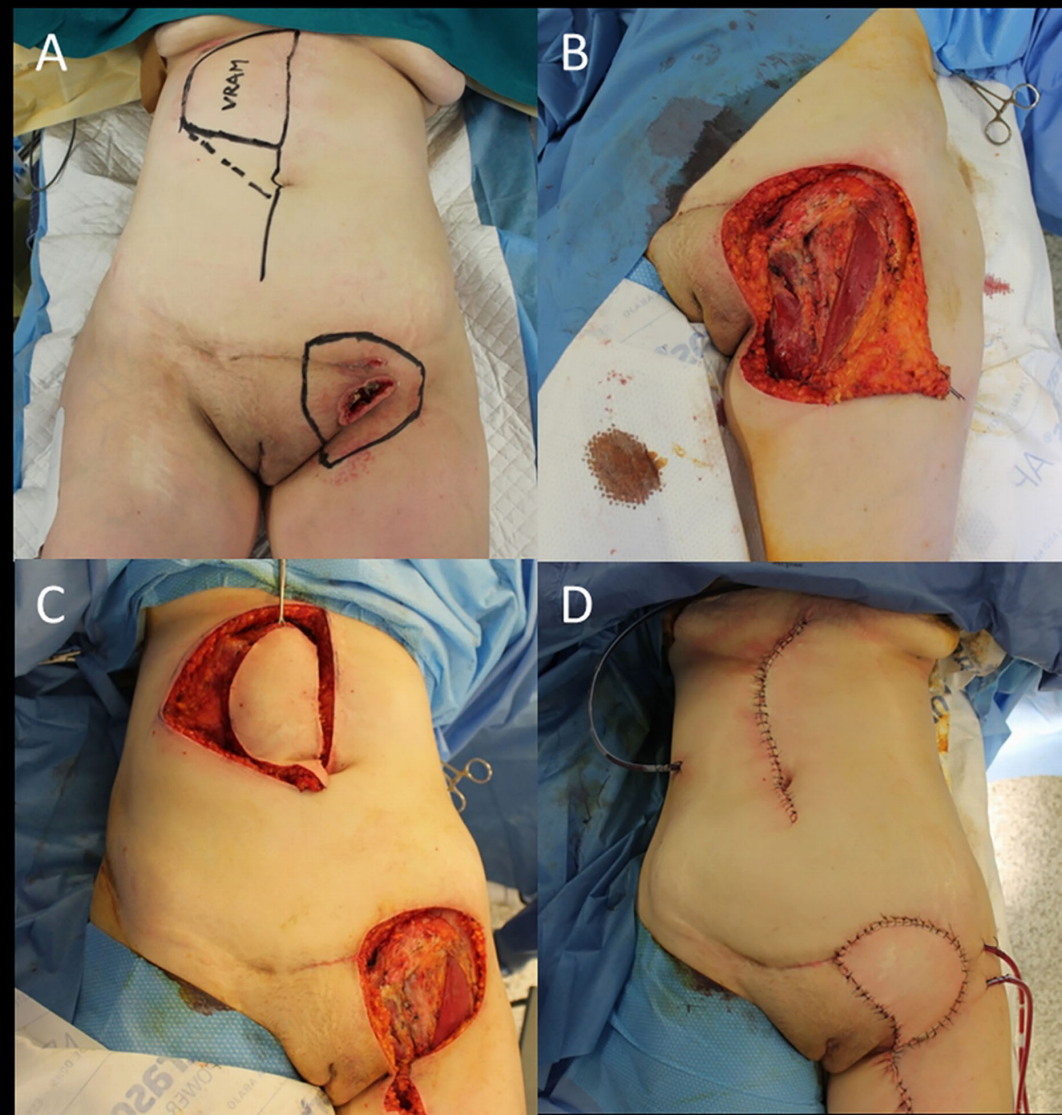
**(A)** Recurrent cSCC of the vulva after surgery and radiotherapy, outlined the groin excision limits and the boundaries of the right vertical rectus abdominis muscle (VRAM) flap; **(B)** End of the wide excision and groin dissection; **(C)** Harvest of the VRAM flap; **(D)** Final result after flap rotation.

However, it is not infrequent facing with a regional metastatic cSCC (i.e., vulvar cancer) that involves all the groin region, whose eradication imposes an extension of lymphadenectomy of the abdominal nodes (**Figure 2**).

The structures to be electively spared are the femoral vessels and nerve, some advocate the great saphenous vein saving in order to reduce the probability of subsequent lower limb lymphedema, a side-effect much more frequent than in the upper limb.

The lateral femoral cutaneous nerve, a sensitive nerve, usually cannot be spared due to its subcutaneous course into the Scarpa’s triangle, leaving a numbness area below the inguinal ligament.

Recently, the application of the laparoscopic approach to groin dissection has proven to be safe for oncology and has consistently reduced both complications mentioned above (85).

### SCC Metastases to Special Locations

In addition to anatomically well-defined regional lymph nodes, other sites may harbour cSCC metastases (86–89). These unusual sites may be identified by imaging while staging or as an incidental report.

The surgical management is primarily driven by the necessity of histological diagnosis for correct tumor staging, then a radical excision may be accomplished as an isolated procedure or as the completion of the regional lymph node dissection.

Once again the multidisciplinary tumor board discussion can support the indication to surgery in case of an invasive procedure.

## The Reconstructive Plan

### The Oncoplastic Approach in Skin Cancer

Historically the plastic surgery has found the most brilliant application in head and neck reconstruction after cancer resection, due to the imperative necessity to provide an immediate repair of crucial anatomical structures.

With the progressive improvement of reconstructive methodology together with the increasing demand of better outcomes in terms of functional and aesthetic recovery, to date, a comprehensive skin cancer treatment should include adequate procedures to let the patient returning to a normal life.

For a better planning of which solution would be the best choice to adopt, in other fields of tumor surgery the plastic surgeon is a component of the team that evaluates preoperatively the patient.

In breast cancer units, for example, from the very beginning of the entire care the patient undergoes to plastic preop assessment to early delineate the forthcoming procedure.

The term oncoplastic surgery indicates this special surgical approach to the issue, both oncologic and reconstructive, and the surgical techniques applied to (90).

The necessity of a “skin oncoplastic” approach is not yet be suggested, but the tremendous implications of an aggressive surgery, as required with a locally advanced cSCC, request a redefining of priorities and competences (91).

### Timing of the Reconstruction

Theoretically, the optimal reconstructive procedure would immediately follow the skin cancer excision, to repair the damaged tissues or replace the missing ones.

That point of view is the most favored by plastic surgeons, in regard to better conditions of local residual tissues, less alterations due to inflammatory process and the frequent availability of vessels in the surgical field as source for microsurgical transplant.

An immediate reconstruction requires a fine preop planning, may lengthen of several hours the overall surgery time and may hinder an eventual second look for oncologic purpose because of skin flaps transposition.

The condition that indicates an immediate repair is the incompatibility of the wounds with life or with a reasonable postop recovery, in that case the reconstruction would be mandatory.

Another issue regards the clearance of resection margins, if the ablative extent has been maximum in relation to patient conditions, no matter if definitive histology would report R1 or close margins.

Instead, if the local conditions would permit a widening of resection and the clearance is uncertain, a delayed reconstructive procedure should be seriously considered (50).

The remaining tumor tissue at the edges of the resection will inevitably invalidate oncological and reconstructive procedures, promoting early local recurrence and preventing wound healing.

When free margins are questionable, the most complex reconstructions should be avoided in favor of the less demanding procedures (i.e., skin graft), that may allow temporary and suboptimal repair waiting for histology confirmation.

Today a number of engineered skin substitutes are available (92), mostly derived from porcine or bovine dermal tissue, that consent an immediate defect cover without sacrificing of the patient’s skin.

Another interesting technologies are the vacuum-assisted closure devices (93), a sort of sealing dressing with a permanent aspiration system connected, that may protect the wound from contamination and prepare the surgical bed for definitive repair.

Thanks to these innovative solutions, a delayed reconstruction procedure can be planned safely with minimum patient discomfort and avoiding the problem of margin clearance.

### Functional and Aesthetic Issues

Patients affected by a locally advanced cSCC reasonably will face with a great impairment in quality of life (QOL) as a result of the aggressive nature of their disease leading to extreme surgical procedures (94).

QOL is related to maintaining self-sufficiency, meaning re-establishment of daily activities and vocational rehabilitation, but it is also related to self-esteem.

Age and disease severity may negatively influence QOL, older patients reported significantly lower outcome than younger patients, and a clear reduction of QOL is considerable when patients with NMSC diagnoses are compared to those with actinic keratosis only (4 to 9%) (95).

The concepts of repair and of reconstruction may greatly differ in relation to the final outcome, because promoting the wound healing not necessary means for the patient a return to the preop physical and mental state.

Therefore, the simplest reconstruction will require less time to be accomplished, will be less heavy for the patient, but probably will not fully meet the needs after a complex tumor resection.

For example, the skin graft, probably the most largely used plastic surgery technique, is not free from concerns due to contraction, poor skin matching, and resulting deformity when applied in an aesthetically sensitive area (96).

Due to scars and unpleasant outcomes, most patients suffer some degree of psycho-social distress related to appearance, especially during the short-term post-operative period (97).

Advanced reconstruction skills are often necessary to improve the overall outcome, especially in topographically challenging areas, such as the face or upper extremities.

The correct use of the plastic surgeon’s tools encompassed in the reconstructive ladder may be the key to better functional recovery and satisfactory result.

## Combined Procedures

### Head and Neck

The head and neck area is by far the most common location for primary cSCC.

Historically, that was the first field of application of plastic reconstructive techniques after oncologic resections, due to the impossibility of amputation and the dramatic consequences of the second intention healing when it was achievable.

If the small SCC can be easily cut and repaired by a local cutaneous flap, the locally advanced one poses severe challenges in terms of functional impairment and aesthetic demands.

With the relative exception of the nose, more usually affected by basal cell carcinoma, the chronically sun-exposed areas, such as lips, forehead, ears and scalp, can be largely involved by tumor development requiring full-thickness excision of soft tissues and, not rarely, of the underlying bone surface.

For the forehead and the scalp usually the simplest technique, the skin graft, plays a significant role.

Consists in the harvest of a slice of dermal-epidermal layer of 0.4-0.5 mm in thickness with the use of a mechanical dermatome.

It is possible to take the graft manually with the aid of a blade, but in this case it will inevitably result in a greater thickness.

The biological concept is the possibility to transfer portions of skin to another side without vessel anastomosis, due to the limited amount of tissue cells transferred, that can be successfully supported by the underlying healthy tissue.

The major limit of the procedure consists in the necessity of a viable recipient bed, some tissues like bone, tendon, muscle fascia, and loose fatty tissue may not provide a sufficient blood supply to permit the graft survival.

Recently, a number of templates have been introduced in clinical practice to facilitate the skin grafting, acting as a scaffold for the regenerative tissue towards the graft.

The application limits the collateral shrinkage that usually affects the graft after maturation and permits grafting on uneven surfaces with sub-optimal perfusion.

Two models of regenerative templates are available, the first is expected to be immediately skin grafted, the second is covered by a temporary silicone patch and requires about 15 days before be grafting.

**Figure 3:**
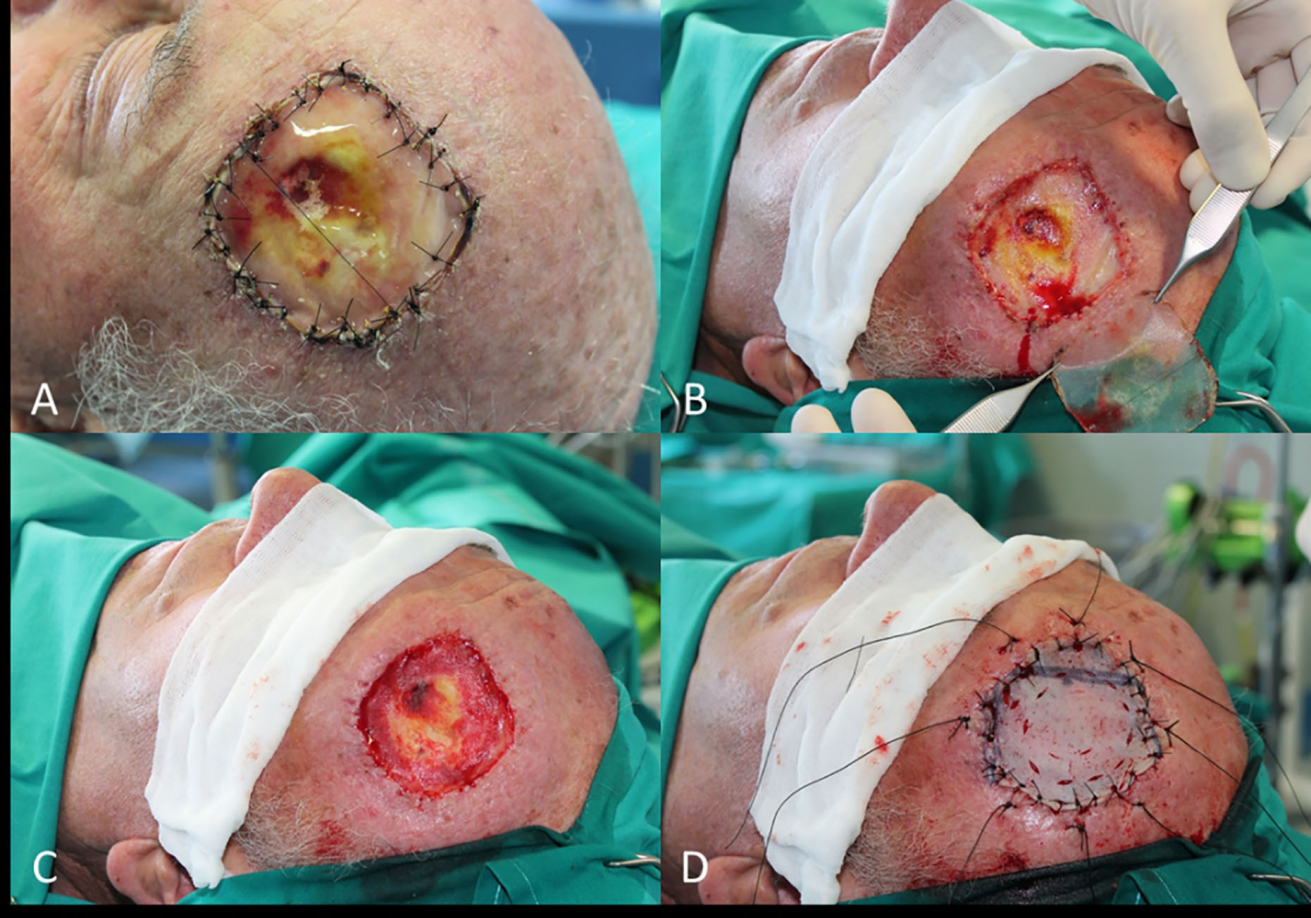
**(A)** Reconstruction of scalp after WLE and regenerate template application; **(B)** Removal of silicone patch; **(C)** Wound bed debridement; **(D)** Final closure with skin graft.

An example of procedure with wide large soft tissue excision and outer calvarian resection followed by two step reconstruction with skin substitute (template) and a skin graft is reported in **Figure 3**.

Instead, the surgical repair with a flap becomes necessary when the local conditions of the wound bed after resection will not allow direct or delayed grafting.

The flap has the great advantage of being independent from wound conditions thanks to its own blood supply.

A further benefit of respecting skin grafts is the quality of reconstruction that flaps can provide.

The wide large excision can leave a dead space to be filled, or may deprive bone of the essential soft tissue cover, in such situation only a reconstruction with a flap can be successful.

It requires a surgical dissection, so it is a time-consuming procedure and the area of the body from which it is harvested has to suffer considerable damage.

Undoubtedly, with proper flap selection and meticulous technique these disadvantages will be significantly reduced.

Flaps can be variably classified according to the tissue transferred (i.e., skin flap, muscle flap, bone flap), or on the basis of the specific blood supply (random flap, pedicled flap, island flap, perforator flap).

The first choice in the reconstructive procedures ladder will be a local flap, due to the proximity of the defect, and the low impact on the patient.

**Figure 4:**
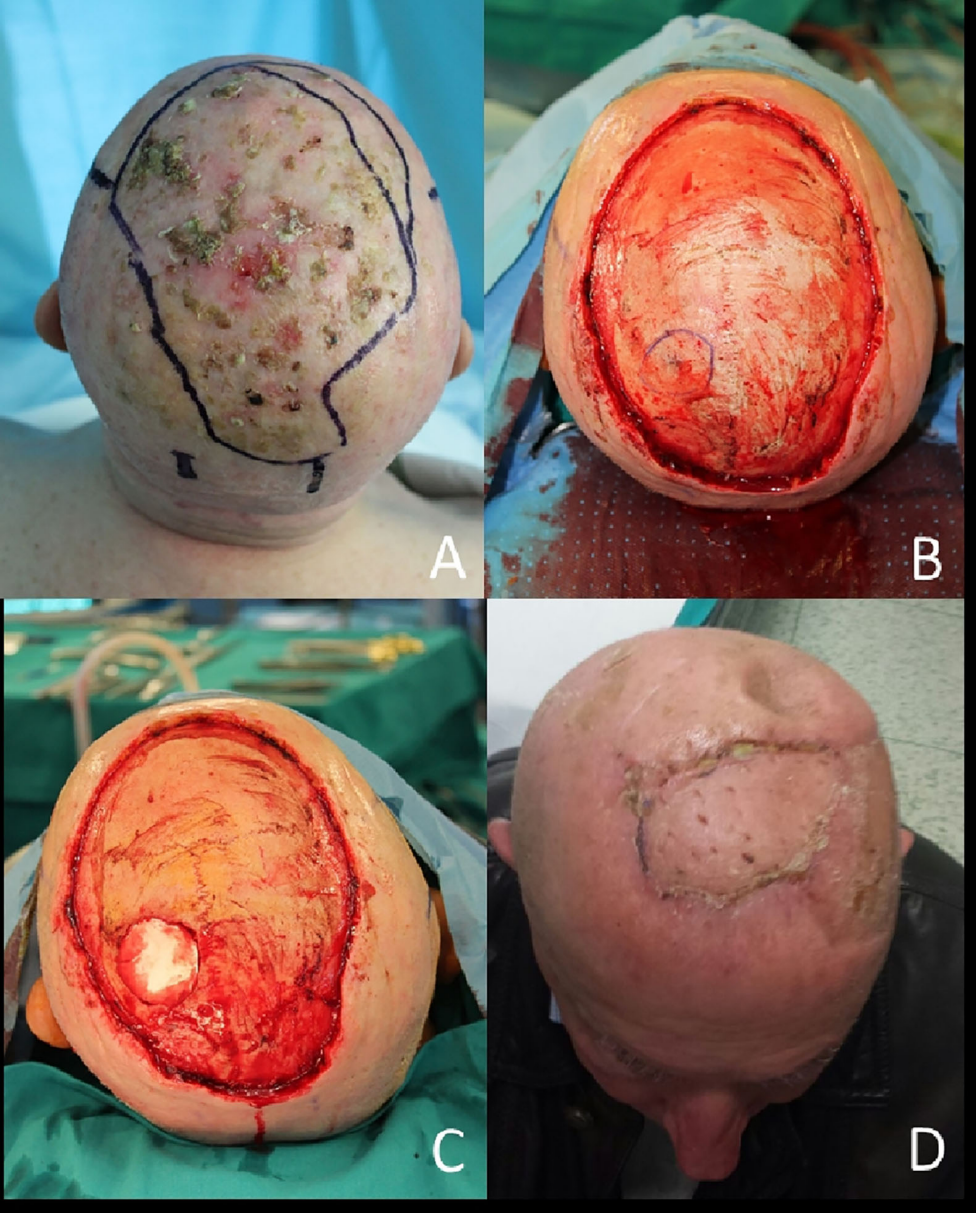
**(A)** Field of cancerization on the scalp; **(B)** After en bloc resection, in blue outlined the tumor invasion of calvarian; **(C)** Bone resection complete; **(D)** Final result with local flap and skin grafts at 1 month.

To replace a soft tissue defect usually a random skin flap is sufficient, but when the defect is too wide (i.e., a cancerization field) a multimodal flap-based reconstruction and skin grafts will get the result (**Figure 4**).

**Figure 5:**
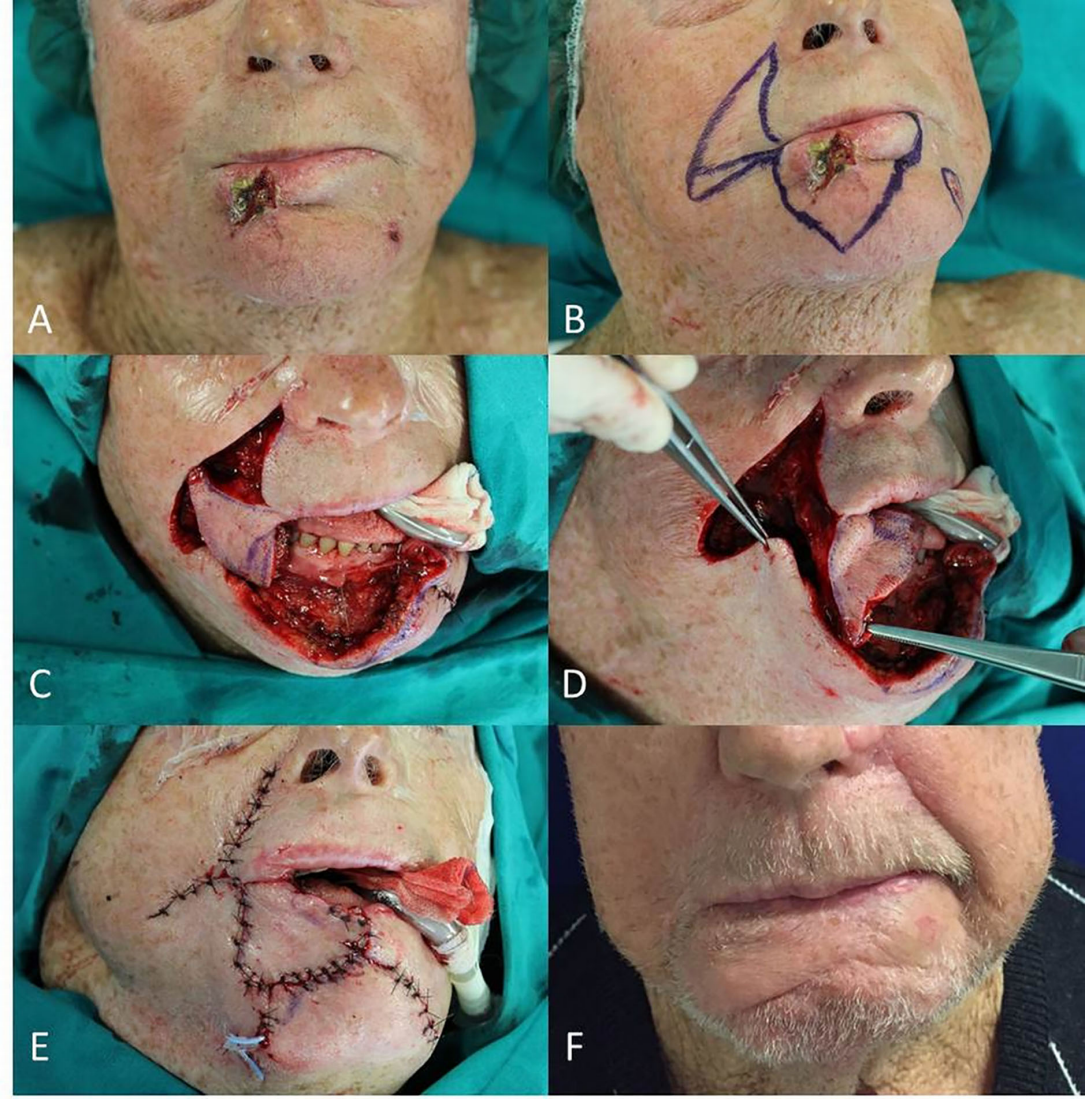
**(A)** Locally advanced SCC of the inferior lip; **(B)** Planning of the wide wedge excision and the Estlander flap from the upper lip; **(C)** Soft and hard tissues removed; **(D)** Setting of the flap pedicled on the superior labial artery; **(E)** End of surgery; **(F)** Follow up at 6 months after right commissuroplasty.

**Figure 6:**
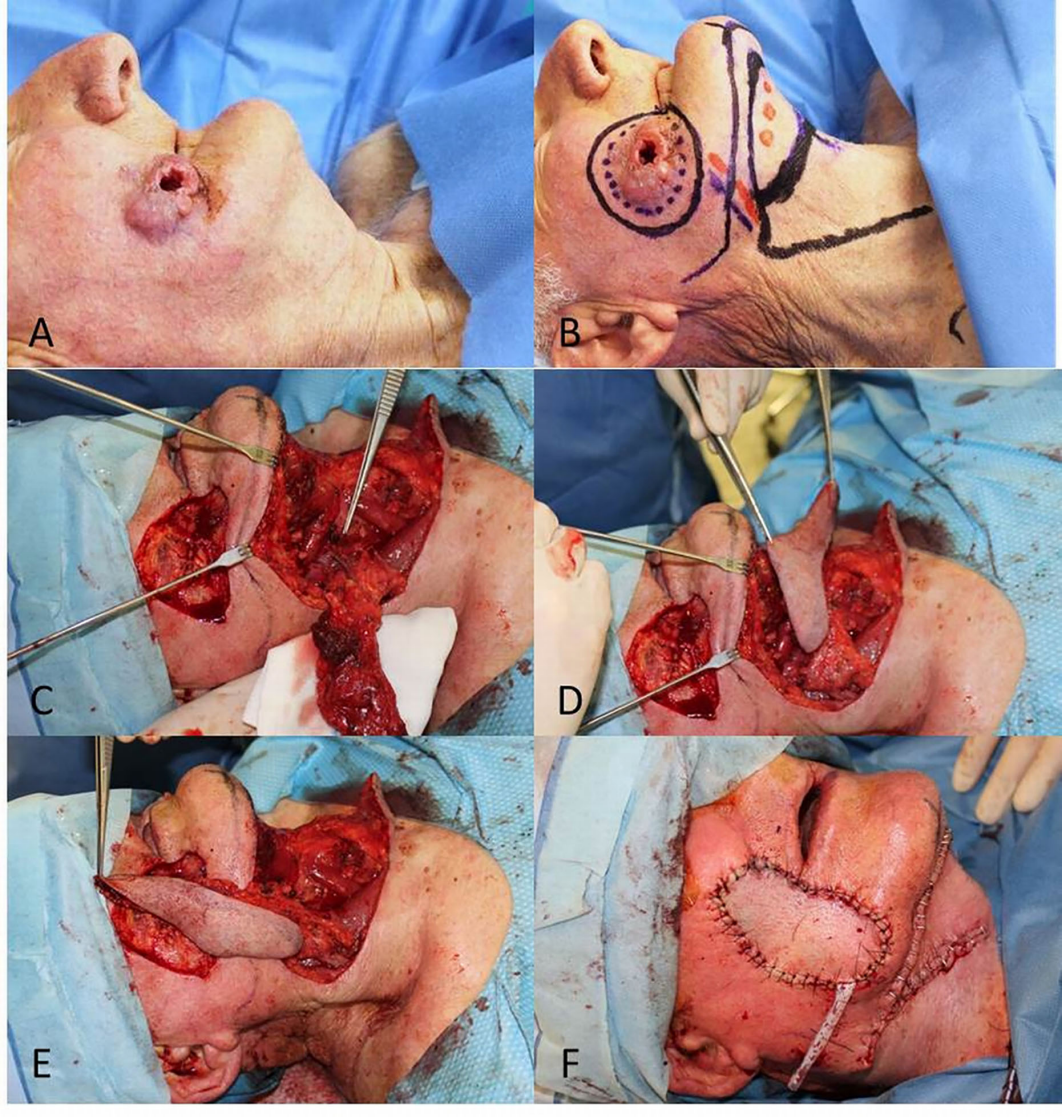
**(A)** Locally advanced SCC of the cheek; **(B)** Planning of the full-thickness excision and the submental flap; **(C, D)** Excision complete and dissection of the flap based on the submental vessels; **(E)** Advancement of the flap to the defect; **(F)** Final result.

Another indication for performing flap-based reconstruction is the need for functional recovery when the whole anatomical subunit must be excised. For example, when a loaSCC arises in the lips a wide wedge resection will not permit the direct approximations of the three-layers structure of the lip, that will cause the inability in maintaining the bolus inside the oral cavity during eating. A pedicled local or regional cutaneous flap (**Figures 5**, **6**) , turned into the defect, will provide soft and elastic tissue that will act as a “bridge” to restore the oral boundaries and its sealing properties.

Moving from medial to lateral, the pre-auricolar region and the ear presents some of the most challenging problems to solve in case of a locally advanced SCC.

The presence of several different tissues and anatomical structures within a few centimeters (skin, muscle, bone, parotid gland, facial nerve, outer and inner auditory canal) often require extremely wide resection due to the tumor quick in-depth grow.

**Figure 7:**
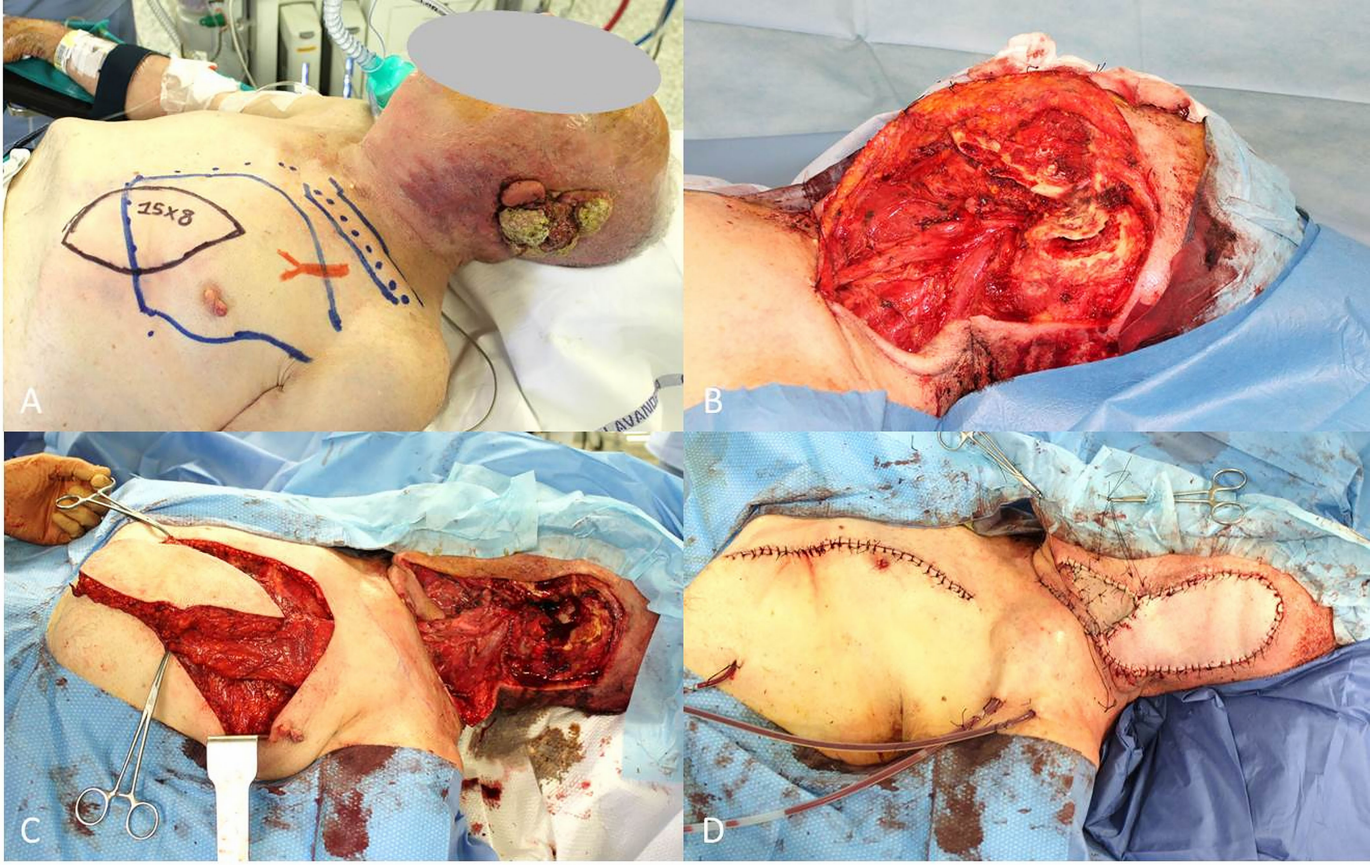
**(A)** Locally advanced SCC of the ear and planning of pectoralis major muscle flap; **(B)** End of petrosectomy and neck dissection; **(C)** Pectoralis muscle flap harvested; **(D)** Final result after flap rotation.

The major pectoralis flap is a workhorse that has been employed for decades and still represents the “plan B” after failure of more sophisticated flaps or when the patient cannot sustain a time-consuming procedure (**Figure 7**).

More recently, the free flaps have replaced the pedicled ones, such as the major pectoralis flap, deltopectoral flap and Trapezius muscle flap.

Pedicled flaps are limited in the rotation by the length of the nourishing artery and concomitant vein, if the defect lies too distally from the blood supply another solution must be identified.

The ultimate, most complicated, technique of plastic surgery is the microsurgical free flap, which theoretically can provide healthy tissue in any part of the body.

The basis of microsurgical transplantation is the transfer of a part of the body (skin, muscle, bone, nerve or a combination of) by a vascular microanastomosis performed under a magnification microscope.

**Figure 8:**
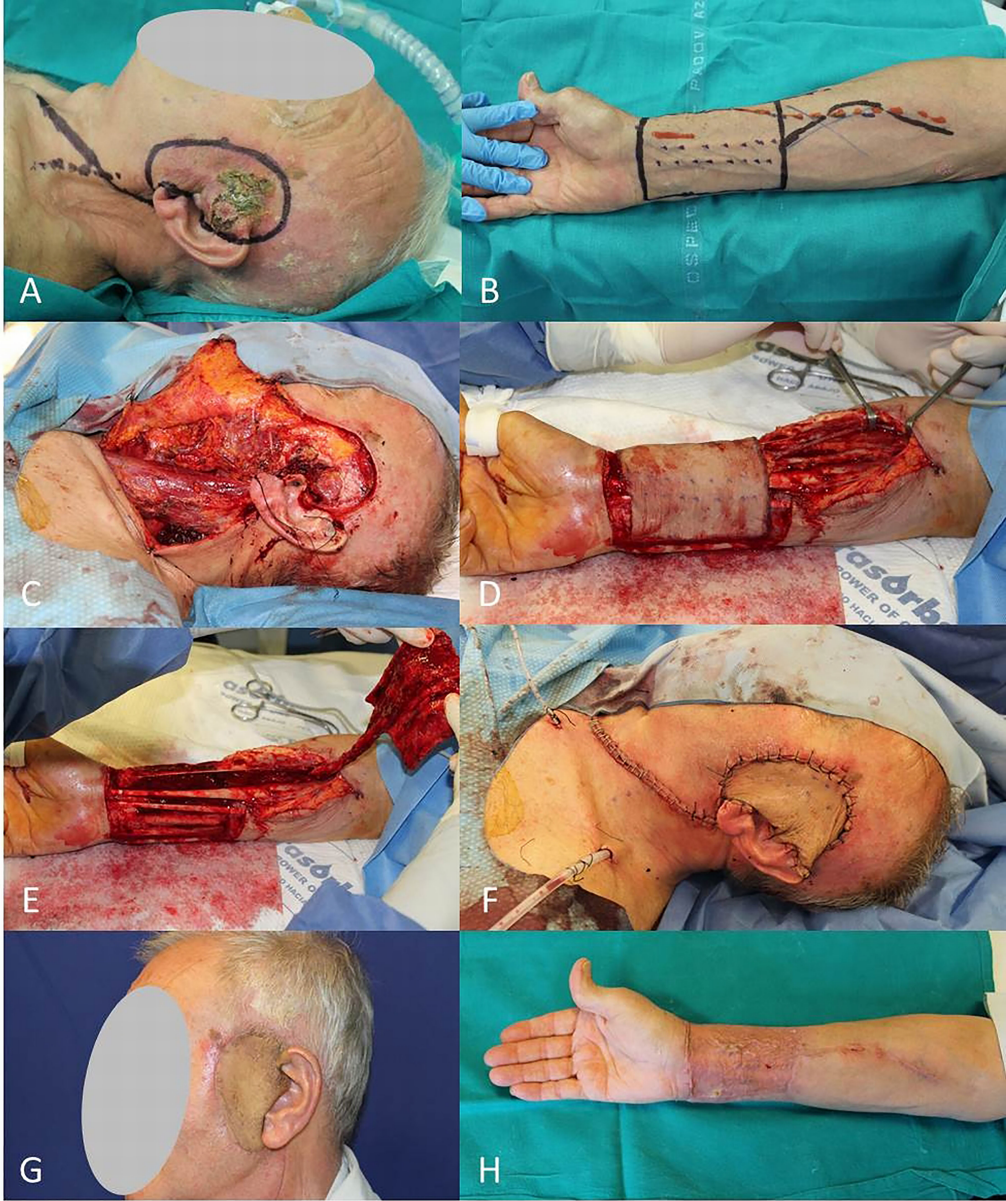
**(A)** SCC of the preauricolar area; **(B)** Radial antebrachial free flap planning; **(C)** End of WLE and neck dissection; **(D, E)** Harvest of radial flap; **(F)** Immediate final result; **(G, H)** Free radial flap and donor site after 2 months.

**Figure 9:**
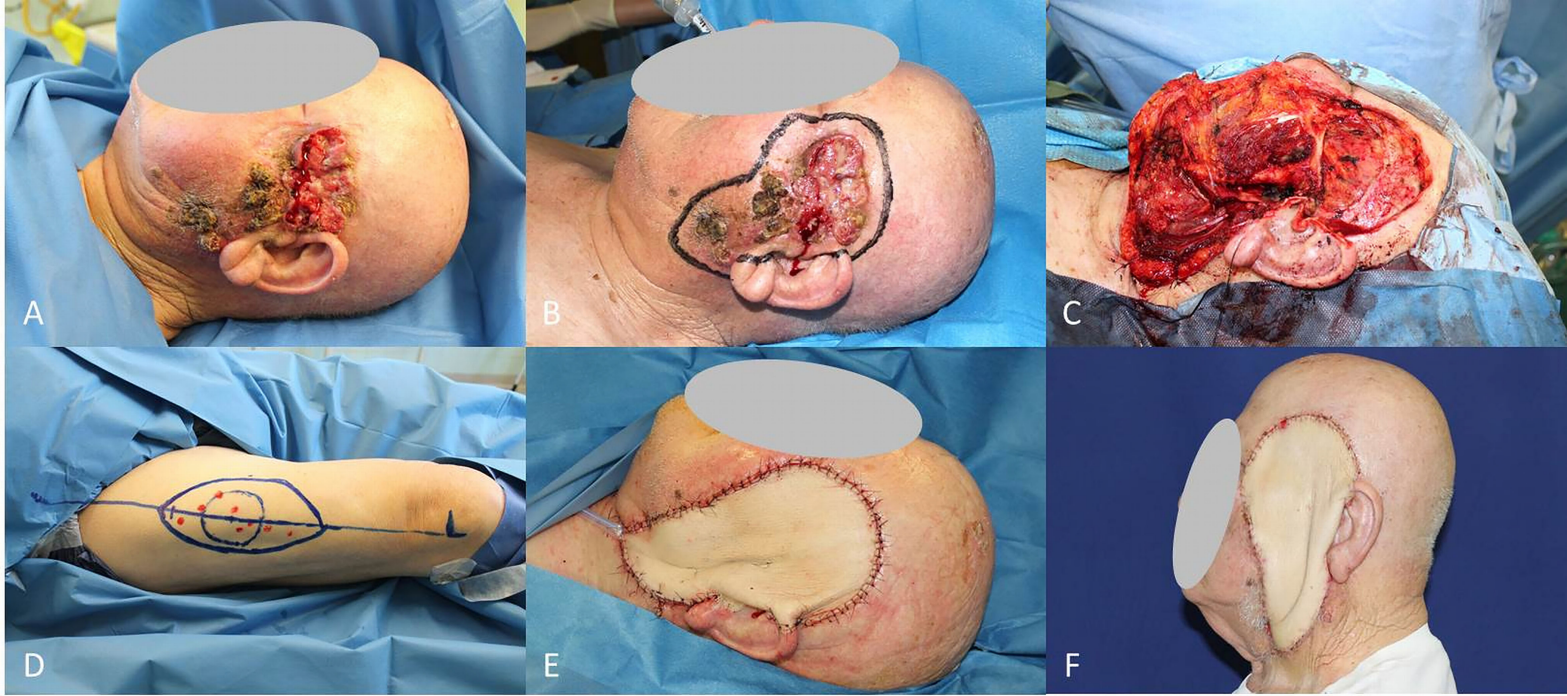
**(A)** Locally advanced SCC of left midface; **(B)** Planning of the wide excision; **(C)** Soft and hard tissues removed, lymphadenectomy completed; **(D)** Planning of the ALT flap harvest; **(E)** Immediate result after the ALT flap insetting; **(F)** Final result at 30 days follow-up.

Free flaps have been harvested from every part of the entire body surface, like the upper limb (i.e., radial or Chinese flap, **Figure 8**), the back (i.e., latissimus dorsi free flap), the trunk (i.e., deep inferior epigastric artery perforator flap - DIEP and superficial circumflex iliac artery perforator flap - SCIP), the thigh (i.e., anterolateral thigh flap – ALT, **Figure 9**), and the leg (i.e., medial sural artery perforator flap - MSAP).

Microsurgery has proven to be an efficient and reliable tool, making it the preferred choice for many oncology reconstructions (98).

The advantage of the free flap technique is the possibility to choose the tissue to transfer in the base of the necessity of the single case, replacing the missing tissue with one analogous with the same proprieties and characteristics.

These complex reconstructions require several hours, a dedicated operating room setting and high qualified personnel.

The overall quality of the outcomes with free flaps are largely superior than with conventional techniques (graft and random local flaps), the functional recovery is higher and faster, and the reconstruction aesthetic, whenever possible in these cases, is much better.

In most cases the microsurgical transplant provides the only real chance to perform extremely large and aggressive oncologic resections, otherwise impossible, therefore, has to be intended a part of the comprehensive tumor treatment.

### Trunk

The trunk is a less common site of loaSCC development, and the conventional repair with direct tissue approximation after extensive subcutaneous undermining is straightforward most of the time.

When instead the anatomical region has to be necessarily restored, like in the case of a radical vulvectomy for loaSCC of genitalia, the surgeon has to turn to a flap-based reconstruction.

The progression of anatomical studies on the soft tissue vascular supply has led to the development of skin flaps based on a single vessel coming from the underlying layer, the so-called perforator flaps.

The versatility and minimum sacrifice associated with these flaps extended the scope.

Dissection can be much more tedious, because of the need to save other functional structures during the harvest of flaps.

**Figure 10:**
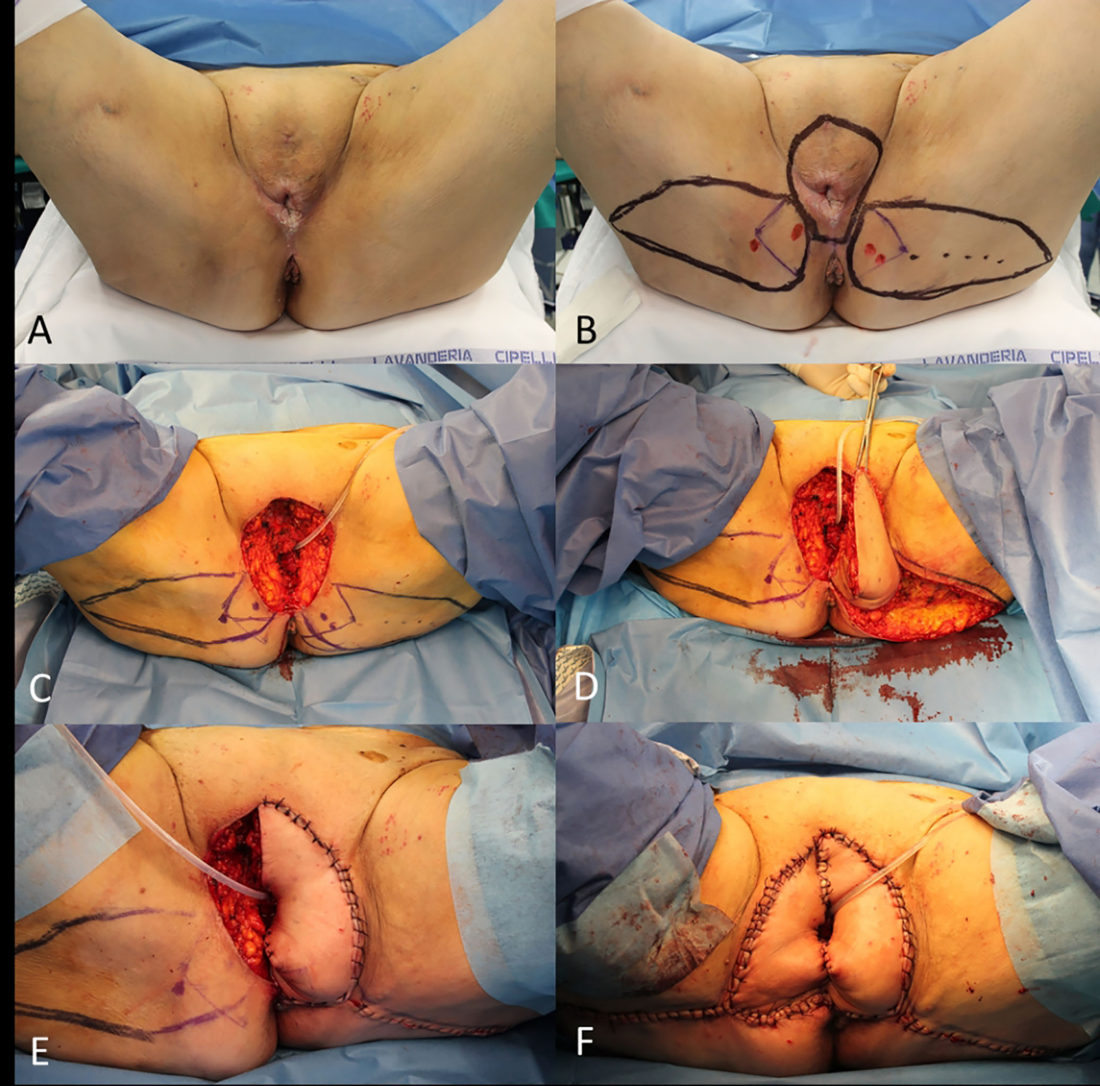
**(A)** Recurrent cSCC of the vulva; **(B)** Planning of the radical vulvectomy and bilateral perforator (lotus) flaps; **(C)** End of the vulvectomy; **(D)** Rotation of the left perforator flap; **(E)** Left lotus flap insetting; **(F)** Final result.

In the reconstruction of female genitalia the use of perforator flaps has replaced in many cases the need to harvest a muscle flap (**Figure 10**).

A muscle flap, in which an entire muscle belly is harvested from its native bed and rotate to cover a defect, still plays a role when no other easier solution is available.

**Figure 11:**
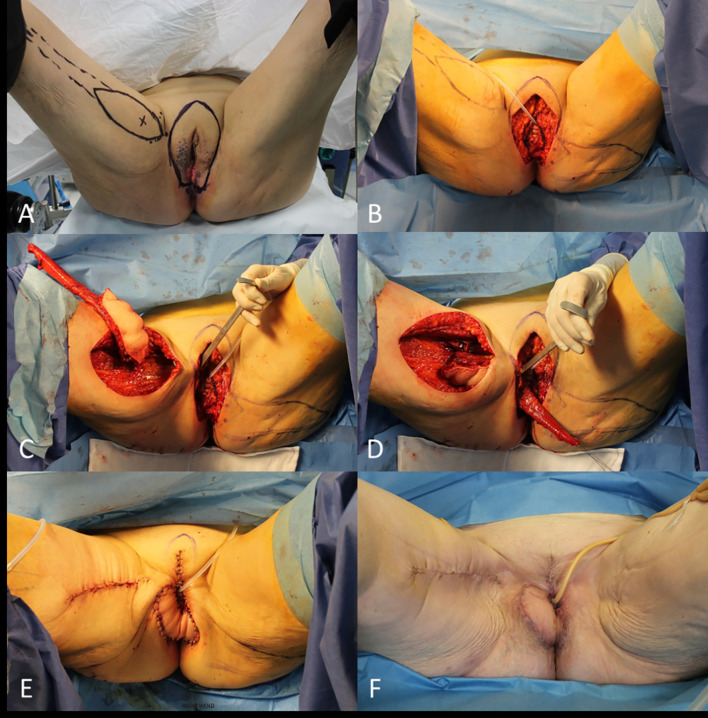
**(A)** Recurrent cSCC of the vulva and planning of gracilis musculocutaneous flap; **(B)** End of the radical vulvectomy; **(C)** Flap dissection; **(D)** Flap transferring; **(E)** Immediate final result; **(F)** Final result at 1 month.

The transferring of a muscle flap with a portion of overlying skin is defined musculocutaneous flap, it permits to fill a deep dead space and to replace the skin cover at the same time (**Figure 11**).

### Upper Limb and Hand

The upper extremities are not often affected by loaSCCs, on the contrary the hand, due to the permanent sun exposure, may be suffering from a rapid-grow SCC, that rapidly impairs the function and causes acute pain due to direct involvement of nerves.

Of course the finger amputation still plays a role, but, in the presence of thumb or multiple digit involvement by tumor, the conservation of a minimal function of grasp is an issue to be addressed.

**Figure 12:**
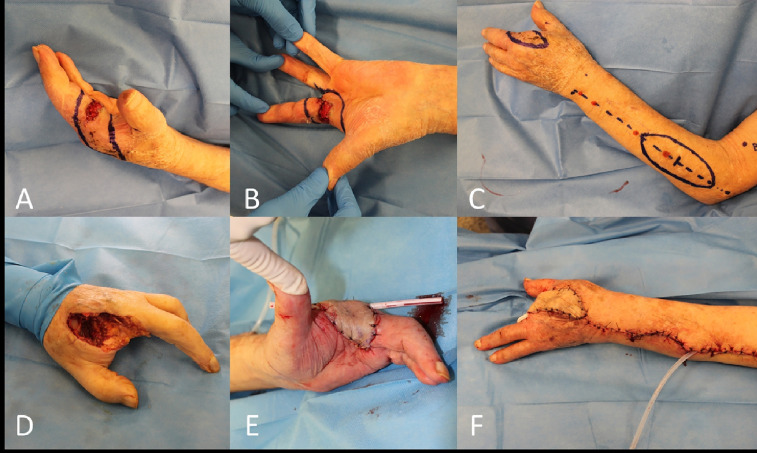
**(A, B)** Recurrent and locally advanced cSCC of the base of the long finger (second amputated previously); **(C)** Posterior interosseous fascial flap planned; **(D)** End of the third hand ray amputation; **(E, F)** Final result after rotation of the flap.

In these selected cases a distant pedicled flap reconstruction should be considered, that may be accomplished through the sacrifice of a major upper limb vessel (i.e., radial flap) or with a less demanding flap like the posterior interosseous flap (**Figure 12**).

### Lower Limb and Foot

The lower limb, in particular the anterior surface of the leg, is often the growth site of cSCCs, many of which can be easily removed and repaired with a skin graft.

The presence of a loaSCC instead invariably requires partial or total resection of a bony tibial tract, making direct grafting impossible.

**Figure 13:**
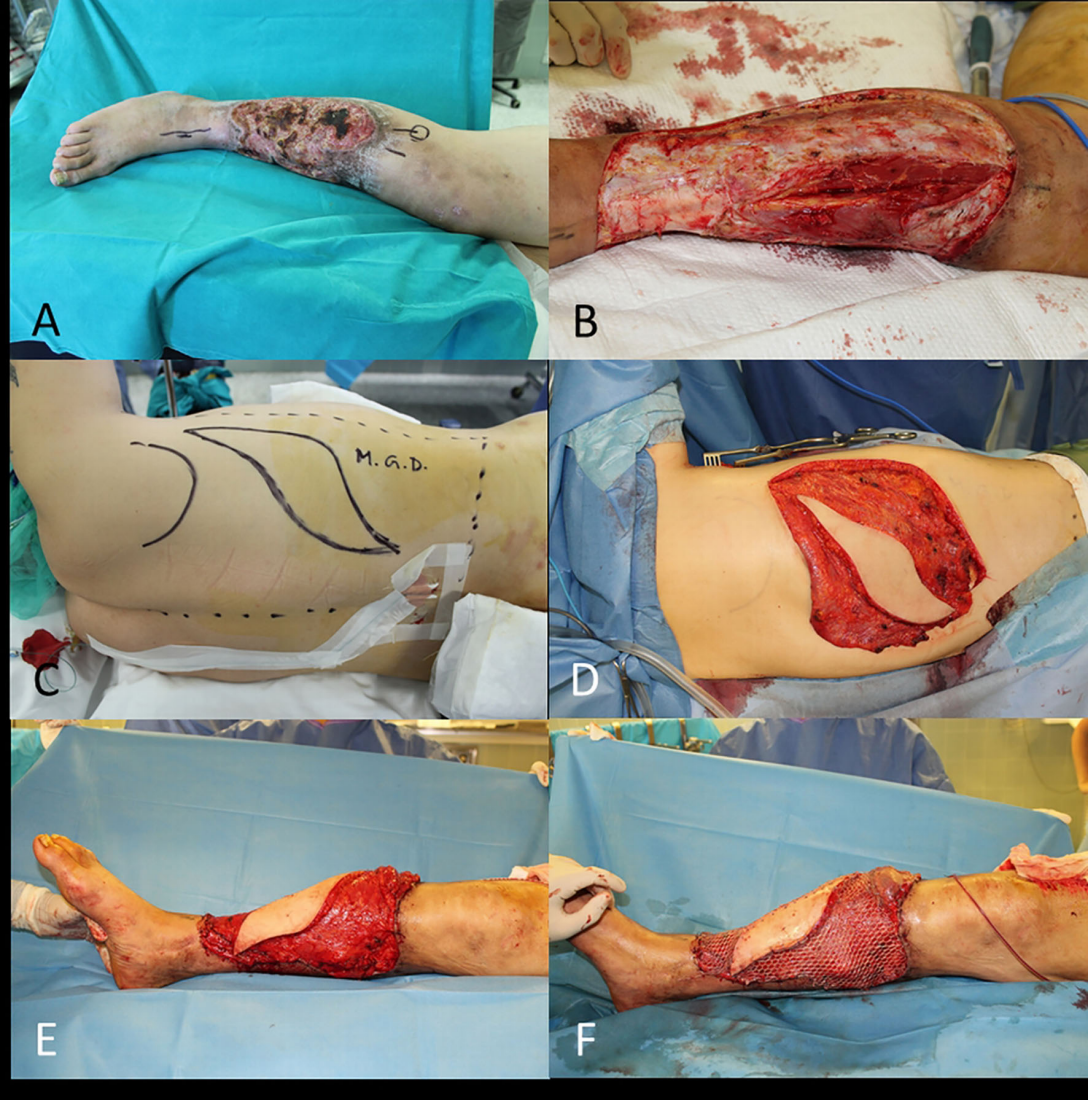
**(A)** SCC of the leg; **(B)** End of WLE with bone exposure; **(C)** Free latissimus dorsi flap planned; **(D)** Flap ready for transfer; **(E)** End of microsurgical transplant; **(F)** Final result with skin grafts.

In this circumstance it is necessary to use a flap, which may be a pedicled flap (i.e., gastrocnemius flap, pedicled MSAP flap, reverse ALT flap), or it will be necessary a microsurgical transplant of a distant healthy tissue to cover the lower limb defect (i.e., latissimus dorsi free flap – **Figure 13**, ALT flap, gracilis free flap).

As for the hand, the amputation is a procedure that has to be considered, especially when it can be safely performed leaving undisturbed about 15cm of the proximal tibial shaft, the minimum length required to be prosthesized.

## Limits of a Surgical Approach

Even the most aggressive excision can cause inadequate surgical margins to guarantee a long-term disease-free period.

The histology features that usually characterize a locally advanced cSCC (size, depth, PNI) have been identified as a risk factor for future recurrence (99).

A history of recurrence, in turn, has been identified as a risk factor for future relapse and poor survival (100).

Therefore, a recurrent cSCC not only represents a local problem, but a high-risk CSC variant that needs to be excised as widely as possible.

A re-excision, even when possible, can be a challenge for the reconstructive surgeon, considering the less available options and scar tissue present.

A second repair with skin graft will permit an early detection of local relapse but functionally and aesthetically can be unsatisfactory.

Another strategy would be delaying the reconstructive procedure, to assess margin status before closing the wound, as suggested above (50).

On the other hand, it is not unusual for recurring cSCC to be caused by multiple suboptimal resections, or close-margin resections, instead of large excision.

This may be due to the impracticability of combined procedures in outpatient settings requiring a tertiary hospital facility.

In this case, after a comprehensive examination of the patient, a more complex procedure may be indicated with the aim of radical resection.

Thus, a review of all reconstructive options is the key to achieve the goal.

It is sometimes the reconstruction procedure that requires a surgical revision due to surgical site infection (SSI) or necrosis of the skin/flaps and dehiscence of the wound.

Replace a necrotic flap with a new healthy one may be challenging, but sometimes is the only reliable “plan B” for salvage procedure (101).

What is not possible in head and neck, it is feasible on the other hand in the extremities.

In presence of a locally advanced cSCC the amputation represents always a choice (102).

In the age of microsurgery may sound inappropriate considering amputation as an option, but older patients affected by numerous comorbidities may be not eligible candidates for such demanding surgery.

More, a reconstructive procedure of a single ray of the hand or a part of the foot may be not compensated by a concrete advantage in term of functional recovery and better outcome, so a frank discussion with patient about realistic pros and cons appears mandatory.

A much less discussed collateral effect of a complex surgery is the impact of anesthetics on immunosuppression in the short-term (103).

A several hours surgery may cause patient debilitation, and anesthetics seem to play a role in transient immunosuppression increase, thus promoting the widespread of cancer not adequately counteracted by the immune system (104).

Again, a careful preoperative evaluation with a multidisciplinary tumor board discussion is revealed as essential for selecting the eligible patients and appropriate treatment.

## Conclusions

The treatment of choice for primary squamous cell carcinoma is surgery, but the locally advanced variant poses a great challenge to obtain free resection margins.

Wide local resection and complex reconstruction are necessary in most cases to fulfil both oncologic and functional requests, although free-disease and overall survival remain uncertain, the patient quality of life may improve considerably.

Risks and advantages for patients undergoing such extreme procedures should be carefully discussed within a multidisciplinary tumor board for better defining patient selection and treatment. In metastatic cSCC and in non-responsive to chemoradiotherapy patients the surgical approach may still play a role in better local control of disease and a salvage procedure.

These challenging procedures are better addressed by a surgical team skilled in plastic surgery techniques in order to provide the best reconstructive options, included microsurgical free flaps.

The preliminary results of the new therapies (i.e., immunotherapy) appear to be very promising, but the relationship with surgery, in terms of timing of administration, is still under investigation.

Further randomized trials are necessary to better define tumor independent factors that impact on overall survival and to compare different multimodal treatment strategies.

## Research Limits and Bias

The literature review was conducted with an on-line research through PubMed^®^ database, inclusion criteria have been data publishing since 2000, articles pertinent to the topic, and full-text available.

For any chapter we performed a dedicated database research combining the terms “cutaneous Squamous Cell Carcinoma”, “cSCC”, “locally advanced cutaneous SCC”, “lacSCC”, “NMSC surgery” with Boolean term “AND” with the specific topic of the sub-section (i.e., imaging, resection margins, radiotherapy, etc.).

Priority was given to studies with a higher level of evidence (systematic review, prospective design) but the majority of the studies presented a retrospective design.

Due to the limited number of available data inherent to some specific aspects of the research field, we included selected case reports and opinion papers in relation to their uniqueness and the marked adherence to the topic.

Besides the research limits some publishing bias has to be mentioned, as the small size of data considered in some articles, the discrepancy in number between study and control groups, and lack of systematic of some reviews.

The above reasons have precluded a robust statistical data analysis, and further randomized trials are strongly recommended before drawing any definitive conclusion.

## Author Contributions

TB, concept and design, data acquisition and analysis, drafting, accountability for all aspects of the work. GA, data acquisition, drafting, accountability for all aspects of the work. PT, concept and design, accountability for all aspects of the work. GM, drafting, accountability for all aspects of the work. AL, concept and design, accountability for all aspects of the work. BB, data analysis, drafting, accountability for all aspects of the work. VV, data analysis, drafting, accountability for all aspects of the work. FB, concept and design, drafting, accountability for all aspects of the work. All authors contributed to the article and approved the submitted version.

## Conflict of Interest

The authors declare that the research was conducted in the absence of any commercial or financial relationships that could be construed as a potential conflict of interest.

## Publisher’s Note

All claims expressed in this article are solely those of the authors and do not necessarily represent those of their affiliated organizations, or those of the publisher, the editors and the reviewers. Any product that may be evaluated in this article, or claim that may be made by its manufacturer, is not guaranteed or endorsed by the publisher.
